# Influence of Breastfeeding on the State of Meta-Inflammation in Obesity—A Narrative Review

**DOI:** 10.3390/cimb45110565

**Published:** 2023-11-11

**Authors:** Dominika Mazur, Małgorzata Satora, Anna K. Rekowska, Zuzanna Kabała, Aleksandra Łomża, Żaneta Kimber-Trojnar, Bożena Leszczyńska-Gorzelak

**Affiliations:** Chair and Department of Obstetrics and Perinatology, Medical University of Lublin, 20-059 Lublin, Poland; dominika.hul20@gmail.com (D.M.); msatoraa@gmail.com (M.S.); arekowska@icloud.com (A.K.R.); zuzanna.kabala00@gmail.com (Z.K.); aleksandra.lomza98@gmail.com (A.Ł.); bozena.leszczynska-gorzelak@umlub.pl (B.L.-G.)

**Keywords:** breastfeeding, obesity, metabolic inflammation, metabolic programming, energy metabolism, immunology

## Abstract

Obesity has become an emerging health issue worldwide that continues to grow in females of reproductive age as well. Obesity, as a multisystem and chronic disease, is associated with metabolic inflammation, which is defined as chronic low-grade systemic inflammation mediated by, i.a., adipose tissue macrophages. Lactation has been proven to have a beneficial influence on maternal health and could help restore metabolic balance, especially in the state of maternal obesity. In this review, we aimed to analyze the influence of breastfeeding on chronic low-grade meta-inflammation caused by obesity. We performed a comprehensive literature review using the PubMed, Science Direct, and Google Scholar electronic databases. For this purpose, we searched for “metabolic inflammation”; “meta-inflammation”; “obesity”; “breastfeeding”; “fetal programming”; “energy metabolism”; “postpartum”; “immunity”; “immune system”; and “inflammation” keyword combinations. While the clinical impact of breastfeeding on maternal and offspring health is currently well known, we decided to gain insight into more specific metabolic effects of adiposity, lipid, and glucose homeostasis, and immunological effects caused by the activity of cytokines, macrophages, and other immune system cells. Further research on the immunological and metabolic effects of breastfeeding in obese patients is key to understanding and potentially developing obesity therapeutic strategies.

## 1. Introduction

Metabolic inflammation (meta-inflammation) is characterized by chronic low-grade inflammation, as opposed to an acute response of the inflammatory system. Macrophages play a significant role as the first line of defense against internal or external dangers and microorganisms by the initiation of inflammatory response. The most common conditions related to meta-inflammation, observed in all tissues involved in the homeostasis of energy, are type 2 diabetes mellitus, overweight or obesity, and metabolic syndrome [1]. Adipose tissue inflammation is initiated by dysfunctional adipocytes, in which apoptosis stimulates inflammatory cytokine responses in macrophages. Adipocyte cell death is a preliminary to many events leading to obesity-associated metabolic diseases, such as cardiovascular diseases, type 2 diabetes mellitus, and certain cancers. Meta-inflammation is mediated by macrophages, which are located, beyond adipose tissue, in the colon, muscles, pancreatic islets and liver [2]. Meta-inflammation in the population of pregnant women may lead to developmental programming in utero and play a role in maternal obesity or gestational diabetes mellitus (GDM). Although there is no adequate evidence that maternal inflammation may be connected to the inflammation in fetal serum, the role of the placenta as an inflammatory mediator in the maternal state of obesity or GDM is highly feasible. This state of chronic, low-grade inflammation, especially in women with excessive content of adipose tissue, may be a direct cause leading to overweight or obesity in the future. Moreover, a higher level of BMI is also linked to delayed initiation of breastfeeding and poor lactation in the early postpartum period [2].

Obesity is defined by the World Health Organization (WHO) as a body mass index (BMI) above 30 kg/m2. It is one of the most crucial public health issues in the world, associated with many physiologic, hormonal, genetic, physical, and also socioeconomic, environmental, and cultural factors [3]. Obesity is one of the most common conditions in women of reproductive age; its prevalence has risen worldwide in the last decades and may lead to many short- and long-term consequences for both the mother and her offspring. Maternal obesity may cause excessive gestational weight gain, leading to cesarean delivery and increasing the risk of development of preeclampsia, GDM, metabolic syndrome, and cardiovascular findings for the mother. The offspring have, among other things, a higher risk of obstetric mortality, many chronic illnesses, and metabolic dysfunction in the future, such as childhood obesity, allergies, and neuropsychiatric disorders. The placentas of obese or overweight women in comparison with normal-weight women have increased levels of total lipid content and a higher accumulation of macrophages and proinflammatory mediators [4]. Maternal obesity is associated with a metabolic, chronic low-grade inflammatory state, due to elevated levels of proinflammatory cytokines., such as interferon γ (IFN-γ), tumor necrosis factor α (TNF-α), interleukin 8 (IL-8), interleukin 6 (IL-6), and c-reactive protein (CRP). Pregnancy itself alters the immune system through changes in placental immune cells for correct implantation, plancentation and growth of a healthy fetus. There is a necessity for balance between pro- and anti-inflammatory cytokines. Hence, prepregnancy obese women are more likely to suffer from infertility, spontaneous pregnancy loss, and preterm delivery [5]. Moreover, increased maternal BMI levels are associated with a higher risk of GDM development, which leads to delayed onset of lactogenesis II and impaired breastfeeding initiation. Pinheiro et al. discovered that, there is an addictive interaction between maternal prepregnancy overweight or obesity and delayed initiation of breastfeeding. Poor lactation in overweight or obese women could be triggered by an abnormal development of mammary glands, cesarean section, preterm birth, fetal macrosomia, and many hormonal or metabolic alterations caused by excessive BMI [6].

The WHO recommends exclusive breastfeeding, which means feeding the child only breast milk, for up to 6 months, and introducing complementary feeding afterward, with additional breastfeeding until their second year of life [7]. The composition of proteins, carbohydrates, lipids, and other breast milk components during two years of breastfeeding varies to provide the energy requirements and guide the intestinal microbiota and immunological system of the infant. Immunologically, breast milk contains cytokines, high levels of antioxidants, and growth factors [8]. This model of nutrition plays a significant role in the child’s optimal growth via enhancing psychomotor and cognitive development as well as decreasing the risk of infectious and allergic diseases and the prevalence of metabolic disorders in the future [9]. Breastfeeding also has positive effects on the mother’s health by reducing the risk of obesity, metabolic syndrome, type 2 diabetes, cardiovascular events, and ovarian or breast cancer [10]. 

Because of the recent attention given to this topic in numerous studies, the impact of breastfeeding and the independent effects of overweight or obesity on maternal and offspring health are currently well known. We aimed to summarize the current state of knowledge about the mutual dependence of breastfeeding and obesity, their influence on chronic low-grade metabolic inflammation, and the short- and long-term consequences of this state.

## 2. Methods

The purpose of this article was to describe the influence of breastfeeding on the state of chronic low-grade meta-inflammation caused by obesity. A review of the scientific literature was carried out without a time limit until October 2023 using the PubMed, Google Scholar, and Science Direct databases.

**Figure 1:**
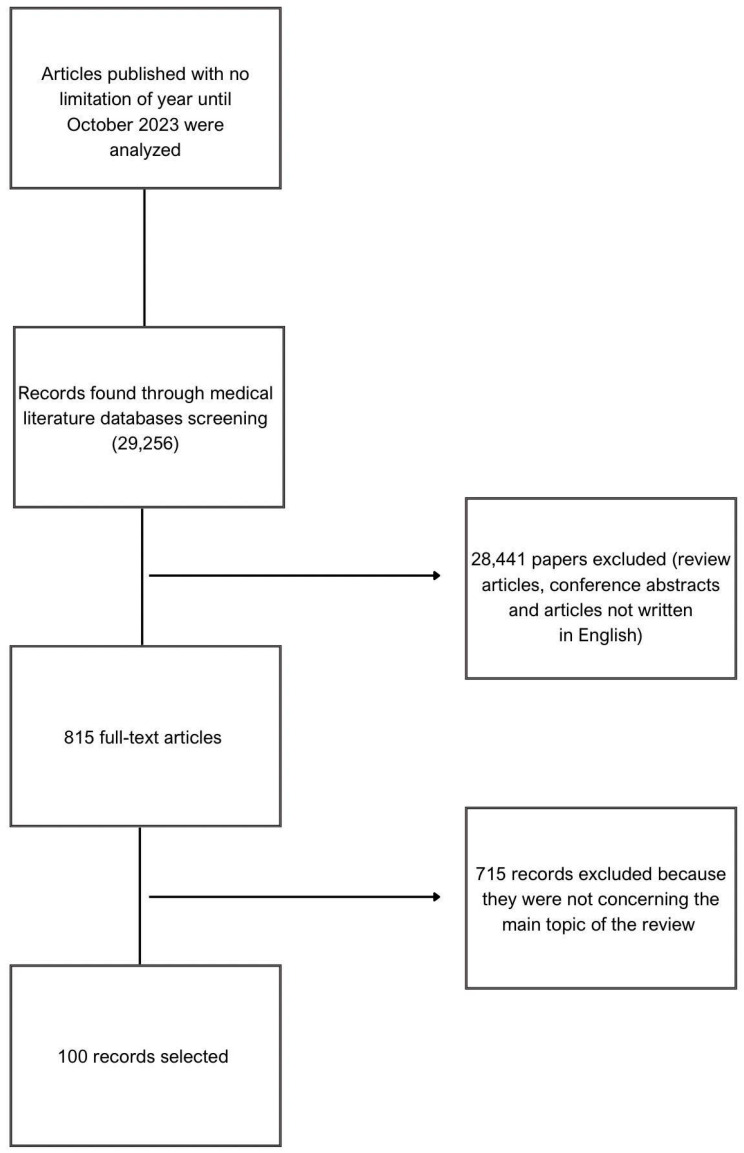
Search criteria for articles used for this review.

Keywords such as “breastfeeding”, “obesity”, “metabolic inflammation”, “metabolic programming”, “energy metabolism”, “immunology”, “postpartum”, “immunity”, “immune system”, and “inflammation” were used to find articles that met the goals of this review. The inclusion criteria for the papers were the following: original papers, retrospective studies, and clinical cases related to the subject. Exclusion criteria were review articles, articles not written in English, and duplicated papers. Additionally, abstracts from conferences and articles outside the subject of the review were also excluded. The detailed method of the literature selection is described in Figure 1.

## 3. Meta-Inflammation in Obesity

Despite the increased awareness of patients regarding nutrition, obesity is still a serious problem—according to the WHO, in 2016 alone, 650 million adults were obese [11]. Moreover, obesity is also the most common chronic disease in women of reproductive age [12]. Obesity is associated not only with the risk of other diseases, such as type 2 diabetes, hypertension, or insulin resistance, but also with many metabolic changes taking place in the patient’s body [13]. Obese patients experience chronic inflammation not only in adipose tissue, but also in skeletal muscles, pancreatic islets, the intestines, and liver [14,15,16]. Therefore, in recent years, research has focused on determining the exact mechanisms responsible for the development of these metabolic changes in people with obesity. Meta-inflammation has an enormous impact on the development of other diseases in obese patients. The inflammation present in the adipose tissue of obese people will be different from the inflammation occurring in patients with injuries, cancers, or infections. Inflammation in adipose tissue is rather mild and, through its effects on various mechanisms, can lead to insulin resistance [17].

In this chapter, we therefore summarize the latest research findings focusing on metabolic inflammation in obesity.

Obesity involves the inflammation of adipose tissue, which undergoes numerous transformations. There are currently several hypotheses available in the literature regarding the exact cause responsible for inflammation in adipose tissue. One of them states that hyperplasia and hypertrophy of adipocytes occur first, which then undergo infiltration by macrophages and fibrosis. Adipocytes become enlarged and move away from the vessels, which leads to their hypoxia and thus reduced adiponectin synthesis [13]. In turn, Woo et al. showed that the main cause of inflammation in adipose tissue is the dysfunction of mitochondria located in adipocytes. Mitochondria are involved in the synthesis of adiponectin, which is believed to play a role in maintaining metabolic homeostasis [18]. However, the result of Woo et al.’s study contradicts the adipose tissue hypoxia hypothesis.

Autophagy includes cellular processes whose task is to degrade subcellular molecules (lipids, proteins, organelles) using lysosomes to maintain homeostasis. Research indicates a dysregulation of the autophagy process in adipose tissue, which contributes to the development of obesity, insulin resistance, and diabetes [19]. Regarding adipose tissue, the role of autophagy is crucial in mediating responses through adiponectin and leptin. Gan et al. demonstrated that endoplasmic reticulum stress-dependent autophagy in adipose tissue is inhibited by leptin. Moreover, leptin is involved in inhibiting the induction of autophagy, which leads to the inhibition of inflammatory reactions [20]. Taking into account the fact that leptin activates autophagy in cancer cells, it can also be concluded that an increased level of leptin in the adipose tissue of obese people may contribute to the increased risk of cancer [21].

Macrophages play an extraordinary role in the homeostasis of adipose tissue and are responsible for the development of inflammation. Crown-like structures (CLSs) are groups of macrophages grouped around dead adipocytes that occur in people with obesity. One of the tasks of CLSs may be to weaken phagocytosis. To discuss the role of macrophages in the adipose tissue of obese people in more detail, it seems important to first determine the division of macrophage polarization into M1 and M2. M1/M2 macrophage polarization involves the secretion of functional phenotypes by macrophages in a given microenvironment [1]. Macrophages type II (M2) are mainly found in the adipose tissue of lean people. In turn, adipocytes in the adipose tissue of obese people produce more proinflammatory cytokines, therefore type I (M1) macrophages are dominant in these patients. M1, being activated by cytokines produced by type 1 T helper cells, produces proinflammatory cytokines, including TNF-α, IL-6, interleukin 12 (IL-12) and interleukin 23 (IL-23). M1 is also responsible for producing nitric oxide synthase and the nitric oxide synthase (NOS) isoform of the enzyme, which also has inflammatory properties. M2, in turn, is involved in phenomena that prevent the development of inflammation. M2 is activated by type 2 T helper cells and synthesizes interleukin 10 (IL-10), unlike M1, which produces it in reduced amounts. Moreover, M2 produces reduced amounts of IL-12 and IL-23, which also distinguishes it from M1. Anti-inflammatory cytokines produced by M2 protect against inflammation and the development of diseases such as insulin resistance [1,22]. Studies have shown that adipose tissue macrophages secrete CD11c [23] and CD206 [24]. Macrophages secreting CD11c are associated with insulin resistance and foam cell formation [1]. Moreover, unlike adipose tissue macrophages of lean people, adipose tissue macrophages of obese people contain the CD36 marker [25]. Also important is the fact that the metabolism of macrophages secreting proinflammatory cytokines is related to glycolysis, and the metabolism of macrophages secreting anti-inflammatory cytokines is related to oxidative phosphorylation [26].

Already in 1993, the results of a study by Hotamisligil et al. showed that the level of TNF-α in the adipose tissue of obese rats was much higher than the level of TNF-α in lean rats [27]. Moreover, Weisberg et al. in 2003 proved that TNF-α was secreted by macrophages, the number of which was significantly increased in adipose tissue with weight gain [22]. Russo et al. showed that the white adipose tissue of obese people contains more macrophages than the white adipose tissue of lean people. Moreover, the same authors proved that there are more than two populations of macrophages with specific functions in a given tissue, but their specific number is still unknown [1].

The literature indicates that the adipose tissue of obese patients is characterized by the activation and increase in the number of T lymphocytes, an increase in the ratio of CD8+ T cells to CD4+ T cells, and a decrease in the level of T regulatory (Treg) cells [28]. Regarding B cells, in a 2009 study, Duffaut et al. showed an increase in B cells in the adipose tissue of animals after implementing a high-fat diet [29]. Increased numbers of B lymphocytes can also be demonstrated in patients with type 2 diabetes [30].

Moreover, in obese patients, there is a higher expression of hypoxia-inducible factor, activation of adipocytes, and production of proinflammatory mediators, including interleukin-1β (IL-1β), IL-6, macrophage migration inhibitory factor (MIF), monocyte chemoattractant protein 1 (MCP-10), and reactive oxygen species (ROS). Behind this phenomena stands the insufficient vascularization of hypertrophic adipocytes. In addition, the development of inflammation in obesity is also influenced by processes such as oxidative stress caused by excess free fatty acids and glucose weakening the action of peroxisome proliferator-activated receptors (PPAR), increased death of adipocytes, and, finally, activation of toll-like receptors [15,31].

Another important aspect of metabolic inflammation is the death of adipocytes, characteristic of obesity. A 2008 study showed an increase in dying adipocytes and thus an increase in the expression of proinflammatory cytokines in animals fed a high-fat diet (HFD). Adipocyte apoptosis leads to an influx of immune cells into adipose tissue and the release of damage-associated molecular patterns (DAMPs), which, by activating immune system cells, contribute to inflammation in adipose tissue [32].

The impact of obesity on metabolic inflammation has been extensively described in many studies in both patients and animals. However, it should be remembered that the markers of immune cells in humans will be different from those of immune cells in mice, which should prompt further consideration when it comes to the results of animal studies. Moreover, most studies mainly analyze inflammatory mechanisms in the premental fat pad. Given the fact that depending on its location in the body, adipose tissue will exhibit slightly different inflammatory mechanisms, future research should focus on the characteristics of adipose tissue from various locations. The role of macrophages in obesity has been described in many studies; however, efforts should be made to determine the functions of other immune cells more precisely, including T cells and B cells. Considering the above and the complexity of the immunological mechanisms occurring in obese patients, it seems important to constantly implement subsequent strategies to prevent the development of overweight and then obesity in patients. This will not only minimize the risk of complications related to obesity but also prevent the occurrence of inflammatory changes that affect other tissues in the body and the development of autoimmune diseases or cancer.

## 4. Immunological Changes in the Postpartum Period

Postpartum (puerperium) is the time from childbirth to 6–8 weeks postpartum, during which the maternal body returns to its prepregnancy state [33]. After delivery, when fetal immune tolerance is not required anymore, the maternal immune system undergoes further adaptations, including immune system evolution. A characteristic phenomenon observed in the puerperium is a significant increase in the level of T cells [34]. In general, the postpartum maternal immune system is dominated by Th1 and T17 at the expense of a decrease in Treg. In addition, both specific and humoral responses are augmented postpartum, and the changes persist for up to a year after delivery [35]. Macrophages accumulated in the cervix during labor undergo aggregation and exhibit the M1 phenotype, known as a proinflammatory feature [35]. In the postpartum period, a decreased number of circulating natural killer (NK) and B cells and elevated amounts of circulating T cells are commonly observed [34]. A 12-month-long follow-up on females in the postpartum period revealed decreased NK cytotoxicity up to 6 months after birth [36]. NK cells suppression is observed throughout the pregnancy and several months postpartum, and NKG2A receptors and plasma components could play crucial role in the process [36]. On the other hand, Hidaka et al. reported an increased activity of NK cells in the first month after delivery, along with a transient increase in absolute CD57+ and CD16+ cell counts, which, in principle, are presumed to protect the mother from infections [37]. Decreased CD158a, CD158b, and natural killer cell receptor (NKG2A) expression on NK cells in the first 3 months of the puerperium period was reported by Groer et al. [36]. Components that also increase after childbirth are neutrophils, monocyte chemo-attractant proteins (MCP-1), and macrophage-derived chemokines (MDC) [35]. Significant rises in CD4+ helper cells, Th1, Th17, and Treg cells are observed as well [34]. Christian et al. examined levels of serum proinflammatory cytokines throughout pregnancy up to 4–6 weeks postpartum. Upward trend of IL-6 levels present in pregnancy, grows even stronger in the postpartum period. Interleukin 8 (IL-8), TNF, and interleukin 1 beta (IL-1β) also rose in the puerperium while CRP declined in the course of pregnancy and then after birth. The exception was obese women, who, compared to lean patients, had higher CRP during both pregnancy and postpartum [38]. Giaglis et al. observed that maternal puerperal neutrophils have strong tendencies to form neutrophil extracellular traps (NET) and augmented levels of citrullinated histone H3 (citH3), myeloperoxidase (MPO), neutrophil granulocytes (NE), and reactive oxygen species (ROS) after birth. Gestational NET formation depends on G-CSF, while NET formation after delivery does not and postpartum NETs are tissue factor (TF)-covered [39,40]. A study by Gillespie et al. shows decrease of IL-6, TNFα, and IL-1beta levels postpartum and heading toward early pregnancy values [41].

In the literature, dysregulation of the immune system after childbirth is linked to the occurrence of the first onset of postpartum psychosis and mood disorder [42]. For females with postpartum depression, there was a prominent decrease in all CD3+ cells, CD3+CD4+ T-helper cells, and CD3+CD8+ cytotoxic T cells compared to healthy women in postpartum [43]. In patients suffering from peripartum cardiomyopathy (PPCM), the number of NK cells was lower and number of CD3+CD4−D8−CD38+ cells was higher than in healthy females after delivery [44], whereas patients with postpartum thyroiditis (PTT) had a lower CD4+ T cell subset and CD4+/CD8+ ratio compared to the healthy group at 3 months after delivery [45]. Moreover, immune dysregulation is linked to cardiovascular events and strokes occurring in postpartum following preeclampsia [46].

## 5. Breastfeeding and Metabolic Programming

The term “metabolic programming” refers to the influence of environmental, endogenous, and maternal nutritional factors during the so-called “critical windows of development” (or critical windows) of the fetus in the intrauterine environment (fetal/intrauterine programming) as well as in the early postnatal period. These factors play a role in determining individual development, metabolic changes, and health in subsequent years of life. These factors affect the cellular structure of organs and tissues in fetuses and young children, because of which they play a role in determining individual development, metabolic changes, and health in subsequent years of life [47,48].

The origins of the concept of maternal nutrition’s impact on the fetus date back to the 1930s. The first researcher to delve into this topic was professor of pharmacology Edward Mellanby. In his work, Mellanby observed the influence of a pregnant woman’s nutrition and her diet on the fetus’s health, as well as on several subsequent generations [49]. In the years 1986–1989, British physician and scientist David Barker described the impact of low birth weight on the risk of cardiovascular diseases, obesity, type 2 diabetes, and insulin resistance in adulthood. This was a result of “fetal stress” caused by inhibited fetal growth in the intrauterine environment due to undernutrition, a theory known as the undernutrition hypothesis. Barker’s hypothesis paved the way for further research into the influence of both endogenous and exogenous factors on the fetus and in the early childhood period [49,50,51,52,53,54].

**Figure 2:**
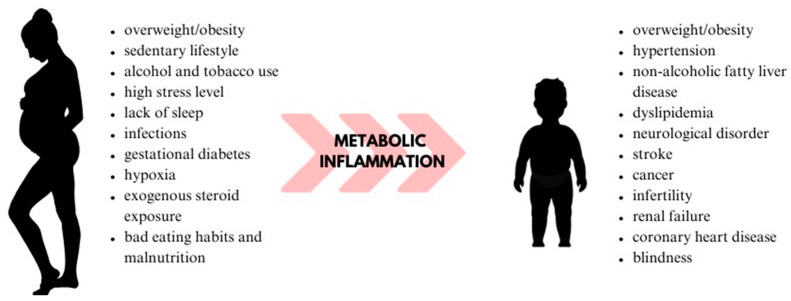
Influence of metabolic inflammation on metabolic programming.

The term “metabolic programming” was introduced by Alan Lucas in 1991. His hypothesis recognizes the impact of unfavorable environmental factors on fetal metabolic programming, leading to chronic physiological changes in the fetus [55]. The “thrifty phenotype hypothesis”, proposed by Hales and Barker in 1992, suggests that a developing fetus exposed to adverse nutritional conditions adapts its organ tissue [56]. This adaptation involves changes in the structure and functioning of cells, including pancreatic beta cells (β-cells), nephrons, and cardiomyocytes. This adaptation involves changes in the structure and functioning of cells, including pancreatic beta cells (β-cells), nephrons, and cardiomyocytes, because of which these mechanisms help the fetus survive in the intrauterine environment. Consequently, this adaptation results in various complications in adulthood. For example, hypoplasia of pancreatic β-cells leads to impaired glucose tolerance (potentially resulting in type 2 diabetes), and a reduced number of nephrons increases the risk of hypertension. Moreover, permanently altered metabolism activates mechanisms responsible for fat tissue deposition after birth, potentially leading to metabolic disorders and obesity [50,51,54,56]. The process of the influence of metabolic inflammation on metabolic programming is shown in Figure 2.

The “fetal salvage” hypothesis posits the development of peripheral insulin resistance, leading to impaired glucose tolerance in the fetus. This mechanism aims to ensure adequate nourishment for essential organs such as the brain, skeletal muscles, and lungs [57]. Among hypotheses related to postnatal weight normalization in children who experienced malnutrition during fetal life, the “catch-up growth” hypothesis stands out. This adaptive phenomenon involves rapid compensatory growth, aligning with genetic predispositions, during the child’s early years. However, this rapid growth is associated with harmful long-term consequences, including peripheral and central obesity, insulin resistance, T2DM, and cardiovascular diseases [58,59,60].

Apart from these mentioned hypotheses, other mechanisms in the literature discuss the influence of nutrition on the fetus. Studies in the field of nutrigenomics, focusing on the interaction between nutrients and genes, describe how supplied nutrients affect gene expression and the body’s metabolic balance. Examples of such interactions include vitamins and minerals that protect deoxyribonucleic acid (DNA) from damage and enhance genomic stability (such as folic acid, vitamins B2, B6, B12, C, E—α-tocopherol, calcium, choline, and magnesium). Their deficiency can lead to DNA breakage and nucleic acid oxidation, potentially causing cleft palate, tumors, and brain dysfunction [61,62]. 

Numerous epidemiological studies confirm Barker’s hypothesis regarding the influence of intrauterine malnutrition (IUM) on the risk of adult-onset diseases. The first study conducted involved the anthropometric observation of 16,000 women and men born in Hertfordshire (UK) between 1911 and 1932. It was found that children with IUM (birth weight < 3000 g) and lower weight in their first year of life were more predisposed to coronary heart disease, the development of type 2 diabetes, and metabolic syndrome. These analyses laid the foundation for the concept that IUM caused by maternal malnutrition during pregnancy could be a risk factor for diseases that occur 60–70 years later [52]. Eriksson and colleagues conducted observations among individuals born in Helsinki (Finland) between 1933 and 1944 regarding the likelihood of developing cardiovascular disease, diabetes, and metabolic syndrome because of IUM and compensatory growth during childhood. Often cited is the study analyzing the health of offspring whose mothers experienced famine during World War II (the Netherlands, winter 1944–1945), when pregnant women faced severe food shortages (<1400 kcal and <1000 kcal/day at the worst period) [63]. These studies demonstrated that the consequences of malnutrition during pregnancy also depend on the fetal developmental stage at which malnutrition occurs. Exposure to famine in the early fetal development stage was associated with an increased risk of coronary heart disease, lipid disorders, clotting disorders, and obesity. Malnutrition occurring in the middle of pregnancy led to microalbuminuria and the risk of obstructive respiratory diseases. If the fetus was undernourished toward the end of pregnancy, it affected the potential for glucose metabolism disorders, significantly increasing the risk of developing T2DM in the future [64,65,66]. Studies describing the health of adults born during the Dutch famine showed increased cholesterol and triglyceride levels, increased body mass index, impaired glucose tolerance, and increased risk of coronary heart disease, and schizophrenia. Similar results were obtained in a study of 40-year-old Nigerians who survived a period of famine (Nigeria, 1968–1970) [64,65,66]. Research conducted among 10- to 12-year-old boys born in Kingston (Jamaica) demonstrated a correlation with high blood pressure in the offspring of mothers who experienced malnutrition during pregnancy [67]. Several studies have been conducted on the protective influence of breastfeeding during infancy and childhood in terms of the occurrence of overweight and obesity in adulthood. It has been proven that each month of breastfeeding reduces the risk of obesity by 4% in later years. This is not only related to a lower body mass index but also an increased HDL fraction in breastfed children [68].

Breastfeeding infants and newborns is the “gold standard” of nutrition, representing the most beneficial way to nourish a young child, ensuring their proper development and optimal health. As a physiological food, breast milk fulfills all of the baby’s nutritional needs. It has excellent biological availability, is easily digestible, and is well tolerated by the baby’s digestive system. The composition of breast milk is always appropriately tailored to the infant’s requirements, varying based on the time of day, the baby’s age, and even during the feeding process. For instance, milk in the initial phase contains more water and less fat than milk in the later phase. Moreover, breast milk is always fresh, at the right temperature, and hygienic. Breastfeeding serves as the best possible preventive measure for a child’s health [69].

Colostrum, the first milk produced by the mother (Latin: colostrum), is the most potent natural immunostimulant known to science. It contains 40 times more biologically active compounds compared to mature milk. Colostrum is rich in passive immunity components for the newborn, such as T and B lymphocytes, macrophages, leukocytes, and antibodies (especially IgA, with concentrations 100 times higher than in mature milk). It also contains highly immunomodulatory elements like lactoferrin (10 times more than mature milk), α-lactalbumin, lactoperoxidase, lysozyme, and casein. While mature milk contains the same immunostimulant components as colostrum, they are present in smaller quantities. Mature milk also contains over 80 enzymes that aid in digesting nutrients, proteins (70% whey fraction, 30% casein), fats (providing 50% of caloric needs), cholesterol (crucial for cell membrane structure and hormone synthesis), vitamin D3, and bile acids (absent in artificial mixtures). The main carbohydrate, lactose, and approximately 100 other oligosaccharides support the gastrointestinal tract’s beneficial bacterial flora. Breast milk also contains various bioactive factors, including immunoglobulins, hormones, and growth factors that promote cell growth and repair. Glycans protect the intestinal lining from pathogenic bacteria, and antioxidants (vitamins A, E, C, β-carotene, glutathione, lactoferrin) safeguard the baby’s health. Breast milk provides nucleotides for DNA construction, as well as blood cells, cytokines, and stem cells. It offers all necessary vitamins, although it is recommended that mothers, especially vegans, supplement their diet with vitamin D3 and vitamin B12. Trace elements (iron, phosphorus, copper, zinc, magnesium, calcium, manganese, potassium, and chromium) are available in breast milk. Iodine and fluoride content depends on the mother’s consumption. Studies have shown that children breastfed by mothers supplemented with 0.3 mg/d of iodine exhibit better psychomotor development [69].

Breastfeeding has an immediate positive impact on the baby’s health, reducing the risk of diarrhea, necrotizing enterocolitis, urinary tract infections, middle ear infections, sudden infant death syndrome (SIDS), bacterial meningitis, respiratory infections, late-onset sepsis in preterm infants, and atopic dermatitis. Long-term benefits include a lower risk of asthma, overweight and obesity, lymphocytic and myeloid leukemia, type 1 and 2 diabetes, non-Hodgkin lymphoma, Crohn’s disease, malignant lymphoma, and breast cancer. Breastfeeding also leads to lower blood pressure, lower cholesterol levels, and a decreased risk of hypertension and/or attention deficit hyperactivity disorder (ADHD) [70,71,72,73,74,75]. Breast milk supports the intellectual and motor development of infants by providing appropriate types of fats that aid in central nervous system development. Studies have shown that exclusive breastfeeding for six months or more enhances intelligence quotient. Premature babies breastfed with mother’s milk exhibit improved motor and cognitive parameters. Sucking at the breast promotes the development of orofacial muscles, which is essential for speech development and prevents jaw misalignment [75]. The emotional bond between the mother and the child is extraordinary, and breastfeeding stimulates all the baby’s senses. Although breastfeeding has numerous advantages, the casual introduction of artificial formula (often influenced by medical personnel) occurs too frequently. Hence, education, awareness, and societal support regarding this matter are incredibly vital. According to the World Health Organization (WHO), exclusive breastfeeding should be continued for the first six months of a child’s life and extended for two years or more [69].

## 6. Breastfeeding’s Impact on the Meta-Inflammatory Process

In a woman’s lifetime, lactation presents one of the biggest metabolic challenges. Several physiological changes brought on by breastfeeding have an immediate impact on the mother’s health and are scientifically proven to lower the probability of the occurrence of many diseases [76,77].

Pregnancy is inextricably linked to the accumulation of energy reserves, expressed in increases in circulating lipid levels, visceral fat, insulin production, and an increased risk of insulin resistance. Lactation accelerates and completes the reversal of pregnancy-associated adverse metabolic alterations [78].

The modification of energy metabolism that occurs in lactating women is aimed at diverting metabolites to the mammary gland, which enables the sufficient caloric enrichment of breast milk. This is possible due to biochemical alterations occurring in multiple organs and tissues, including white adipose tissue, brown adipose tissue, the liver, skeletal muscles, and endocrine pancreas. To provide more fatty acids and glucose for lipogenesis in the mammary gland, the number of fatty acids that the liver, skeletal muscles, white adipose tissue, and brown adipose tissue utilize as a source of energy decreases during lactation.

Likewise, glucose uptake in response to insulin has been proven to be minimized in white adipose tissue, brown adipose tissue, and skeletal muscles, with a high proportion of glycolytic fibers. Moreover, lipolysis in white adipose tissue increases, while gluconeogenesis in the liver and thermogenesis in brown adipose tissue is reduced. Simultaneously with these adjustments, the endocrine pancreas undergoes plastic remodeling, resulting in a temporary loss of pancreatic beta cell mass gained during pregnancy as well as a reduction in insulin output [79]. Bearing in mind all these alterations, lactation’s impact on adiposity, lipid, and glucose homeostasis seems significant, and various research studies have been conducted to prove it.

Rodent studies revealed that in lactating animals, there was a reduction in the amount of lipids stored in adipose tissue [80,81]. Both she size of adipocytes and activity of peripheral lipoprotein lipase (LPL), were decreased in comparison to the non-lactating group [81], and the amount of parametrial fat cells was diminished [82], as was percent body fat [83].

In humans, similar parameters are not that clear to assess because of numerous factors influencing postpartum body mass and body composition [84].

Breastfeeding requires an increased energy cost that adds to the new mother’s overall energy expenditure, raising the possibility of a negative energy balance that might result in weight reduction [85]. According to a study conducted by Butte et al. in healthy women who breastfed exclusively, lactation consumed 2.62 megajoules of energy per day [86]. 

Evidence that nursing can mobilize calories for weight loss comes from a randomized controlled trial conducted by Dewey et al. Four months after giving birth, women who were exclusively breastfeeding were randomly divided into two groups in which they either introduced supplemental foods to their infants or continued exclusive breastfeeding. At 6 months, mothers from the exclusively breastfeeding group had lost 600 g more weight than the remaining subjects, indicating that more intense lactation mobilizes greater adipose storage [87].

Proinflammatory cytokine TNF-α levels are lower, and insulin resistance may be reversed after weight loss [88]. This suggests that breastfeeding can have a beneficial impact on reducing meta-inflammation in obese patients. Therefore, only lifestyle (caloric intake; physical activity) and bariatric interventions have so far been demonstrated to be somewhat successful at counteracting the detrimental effects of obesity and diminishing chronic inflammation, in part by influencing macrophage infiltration and promoting a change from M1- to M2-like macrophages [89]. The role of lactation is yet to be examined. Even the impact of breastfeeding on postpartum weight loss itself remains elusive in larger meta-analyses. Weight change is influenced by multiple factors, including socioeconomic status, ethnicity, prepregnancy weight, gestational weight gain, and lifestyle habits [77]. Frequently, women present a tendency to maintain or even gain weight during lactation [90], presumably as a consequence of their skepticism about caloric intake restrictions when nursing out of concern that it would result in lower milk production [85].

When it comes to assessing the impact of breastfeeding on glucose metabolism, outcomes are more unequivocal. While both pregnancy and meta-inflammation are risk factors for impaired glucose tolerance and insulin resistance, lactation contributes to restoring glucose homeostasis [91].

Regarding animal studies, prolactin, which is physiologically elevated during the breastfeeding period, triggers beta cell proliferation by lowering the amount of menin [92].

More efficient beta cell function was also proven in humans at 3 months postpartum by McManus et al. Fourteen breastfeeding and twelve non-breastfeeding mothers underwent an oral glucose tolerance test (OGTT), a frequently sampled intravenous glucose tolerance test (FSIGT), and computed tomography (CT) to examine insulin sensitivity, glucose effectiveness, and fat distribution. Differences between most variables presented no clinical significance, although in the lactating group, the disposition index was higher [93].

A study by Ozisik et al., including 12 breastfeeding mothers and a control group of 11 non-lactating subjects, exposed an inverse correlation between prolactin, glycated hemoglobin (HbA1c), and 2 h C-peptide [94].

Large cohort studies, both retrospective and prospective, revealed a reduction in the incidence of type 2 diabetes in women who decided to breastfeed their infants [95,96], which proves that the effects of lactation on glucose metabolism are persistent.

The sustained, profitable effect of breastfeeding on β-cell activity was examined both in mice and in humans by Moon et al. The authors hypothesized that prolactin stimulates increased serotonin (5-HT) production in β-cells. 5-HT positively affects β-cell proliferation and insulin secretory function, leading to glucose tolerance improvement. The animal model study proved that assumption and revealed the underlying mechanisms. Subsequently, OGTT results from 85 lactating and 99 non-lactating women were analyzed at two months following delivery and yearly after that. At a mean of 3.6 years postpartum, lactating women presented glucose concentrations comparable to those of 2 months after delivery, whereas in mothers who had not breastfed, a decline in glucose tolerance was noted [97]. Currently, we have limited data concerning the effect of breastfeeding on changes in the level of proinflammatory cytokines.

A murine model study conducted by Roman et al. was designed to evaluate the effects of lactation on metabolic and inflammatory markers. Twelve CD-1 female mice were randomly divided into two equal groups after delivery. The first group nursed pups for 3 weeks and lactated, while the remaining half had their pups removed after birth to avoid the onset of lactation. Markers quantified in serum after 6 months postpartum included IL-6, insulin, leptin, monocyte chemoattractant protein-1 (MCP-1), plasminogen activator inhibitor-1 (PAI-1), and resistin. Results showed an association between lactation and significantly lower levels of maternal PAI-1 and resistin, along with a propensity for lactating mice to have lower levels of leptin. Therefore, other markers remained at similar levels in both groups [98]. 

A study by Ahn et al. on 119 women at 6 months postpartum was designed to investigate the relationship between the stress response, inflammation, and depression symptoms in mothers who primarily breastfeed or bottle-feed their children. While no correlation between breastfeeding and symptoms of postpartum depression was found, the impact of natural feeding on the inflammatory response seemed more notable: IL-6 serum level amounted to 0.76 pg/mL in the predominantly breastfeeding group and was observed to be lower compared to formula-feeding mothers (0.95 pg/mL) [99]. This may imply that breastfeeding has an anti-inflammatory effect.

Another study to support this thesis was conducted earlier by Groer and Davis et al. [100]. The authors discovered reduced IFN- and IFN-/IL10 ratios 4–6 weeks postpartum in breastfeeding mothers [100]. 

However, further research on immune mechanisms is pivotal to more accurately assess and understand the connections between breastfeeding, inflammation and obesity.

## 7. Conclusions

Metabolic inflammation, also known as meta-inflammation, describes a persistent, mild systemic inflammation. This is distinct from the typical transient and immediate inflammatory responses seen in the body’s innate immune system. It is thought to have a substantial impact on the onset of several metabolic diseases. According to current knowledge, macrophages are believed to have a pivotal role in the development of metabolic inflammation. When evaluating the effects of breastfeeding on energy homeostasis, the results seem clear. While both pregnancy and meta-inflammation are risk factors for metabolic diseases, breastfeeding helps restore metabolic balance. Maternal benefits of breastfeeding are linked to limiting the risks of ovarian and breast cancers, osteoporosis, diabetes, hyperlipidemia, hypertension, and myocardial infarction. Breastfeeding also has immediate positive effects on the baby’s health, lowering the risk of conditions such as diarrhea, necrotizing enterocolitis, urinary tract infections, and middle ear infections. It also provides long-term benefits by reducing the likelihood of developing asthma, overweight, obesity, lymphocytic and myeloid leukemia, type 1 and 2 diabetes, and Crohn’s disease. Because of that, women planning pregnancy and expecting a child receive extensive health promotion and health education activities to expand their knowledge about the impact of the mother’s nutritional status on the developing fetus.

Additionally, breastfeeding contributes to lower blood pressure, decreased cholesterol levels, and a reduced risk of hypertension. It is essential to deepen our understanding of the molecular mechanisms behind meta-inflammation, which plays a role in promoting metabolic diseases. This understanding is crucial for developing innovative anti-inflammatory treatments. These therapies should be tailored to individual patients, considering clinical data as well as the specific levels of cytokines, immune cells, and metabolic damage-associated molecular patterns present in each patient.

## Data Availability

Not applicable.
